# Rutin attenuates intestinal toxicity induced by Methotrexate linked with anti-oxidative and anti-inflammatory effects

**DOI:** 10.1186/s12906-016-1069-1

**Published:** 2016-03-10

**Authors:** Raju Gautam, Manjari Singh, Swetlana Gautam, Jitendra Kumar Rawat, Shubhini A Saraf, Gaurav Kaithwas

**Affiliations:** Department of Pharmaceutical Sciences, School of Biosciences and Biotechnology, Babasaheb Bhimrao Ambedkar University, VidyaVihar, Raebareli Road, Lucknow, 226 025 U. P India

**Keywords:** Antioxidant, Immunoregulatory, Proinflammatory, Cyclooxygenase, Lipoxygenase, Cytokines

## Abstract

**Background:**

Methotrexate (MTX) is recognized as an anti-metabolite in cancer chemotherapy and is associated with various toxicities assigned to inflammation and oxidative stress. Rutin has been reported to have significant anti-inflammatory, antioxidant along with antiulcer properties. The present study was undertaken to corroborate the effect of rutin against MTX induced intestinal toxicity in experimental animals.

**Method:**

Six groups of rats (*n* = 6) were dosed with normal saline (3 ml/kg,i.p.); MTX (2.5 mg/kg,i.p.); rutin (50 and 100 mg/kg,i.p.); rutin + MTX (50 mg/kg + 2.5 mg/kg,i.p.); rutin + MTX (100 mg/kg + 2.5 mg/kg,i.p.) for seven consecutive days and sacrificed on eighth day. The intestinal contents were scrutinized physiologically (pH, total acidity, free acidity, CMDI), biochemically (TBARS, protein carbonyl, SOD, catalase and GSH) and for immunoregulatory cytokines (IL-2, IL-4 and IL-10).

**Results and Discussion:**

The administration of rutin demonstrated significant protection against intestinal lesions damaged by MTX. The treatment with rutin elicited noticeable inhibition of free acidity (26.20 %), total acidity (22.05 %) and CMDI (1.16 %) in the experimental animals similar to control. In MTX treated toxic group, the levels of oxidative markers and immunoregulatory cytokines significantly increased in comparison to control, which was subsequently restored after rutin treatment. Rutin also demonstrated 75.63, 81.00 and 80.43 % inhibition of cyclooxygenase-1 and 2, and 15-lipoxygenase respectively.

**Conclusion:**

The positive modulation of MTX toxicity could be attributed to the free radical scavenging and anti-inflammatory (dual inhibition of arachidonic acid pathways) potential of rutin.

## Background

Methotrexate (MTX) is an antagonist of folic acid [1]. MTX competitively inhibits the dihydrofolate reductase (DHFR) enzyme, which participates in the synthesis of folic acid and is requisite for denovo synthesis of purine [1]. Subsequently, MTX is used as an antimetabolite in cancer chemotherapy. Additionally, MTX also plays a crucial role in the treatment of range of diseases including lymphocytic leukemia, non- hodgkin’s lymphoma, osteosarcoma, head neck cancer, and mammary gland tumors [2]. Moreover, MTX is also intervened for treatment of other disease conditions such as rheumatoid arthritis and refractory inflammatory disease [3]. Notwithstanding, the adequacy of MTX is restrained by its relentless and toxic sequel, including intestinal injury, enterocolitis, cardiotoxicity, nephrotoxicity and hepatotoxicity. The MTX administration deteriorates barrier function of the mucosa, resulting in bacterial translocation and inflammation [4]. The intestinal damage include conspicuous morphological injuries in the small intestine, mucosal damage and compromised mucosal barrier function [5]. Pharmacodynamically MTX treatment results in malabsorption, diarrhea, weight loss and disrupted chemotherapy [6]. The mechanism beneath the gastrointestinal toxicity of MTX is below par understood and therefore; the cancer chemotherapy is customarily accompanied by symptomatic therapies such as antibiotic and anti-diarrheal drugs.

Flavonoids are ubiquitous polyphenolic compounds with wide distribution in herbs, vegetables, fruits and beverages [7]. Flavonoids are proclaimed to have antioxidant, anti-inflammatory, anticancer and gastroprotective properties. Flavonoids are reputed to have significant reactive oxygen species (ROS) scavenging along with diminishing effects on arachidonic acid (AA) cascade bagged by dual inhibition of cyclooxygenase (COX) and lipoxygenase (LOX) pathway as well. Rutin is a flavone glycoside, extensively found in black tea, apple skin peel and buckwheat. Antecedently, rutin has been reputed to have significant anti-inflammatory, antioxidant, mucus protective and antiulcer properties through decreased histamine synthesis and increased production of cytoprotective prostaglandins and many more [8, 9]. Rutin has also been reported to strengthen of the capillaries of blood vessels which are the result of its high ROS scavenging activity and antioxidant capacity [10, 11]. In addition, aglycone part of rutin could block both COX and LOX pathway at high concentration, furthermore the lipoxygenase pathway is primarily the target of inflammation inhibition at low concentration of rutin [10, 12].

Considering the role of oxidative stress in MTX associated intestinal damage and unchallenged ROS scavenging activity of rutin, it is hypothesized that rutin can ameliorate the oxidative damage induced by MTX and can be set forth as an adjuvant in MTX chemotherapeutic regimens. Henceforth, the current study was carried out to validate the above hypothesis.

## Methods

### Animals

Wistar strain of albino rats (120–140 g) were procured from the central animal house. Animals were kept under controlled environmental conditions in polypropylene cage at room temperature (25 ± 2 °C) with 12 h light/dark cycle. Animals were fed with standard laboratory animal feed and water *ad libitum*. The experimental protocol was endorsed by institutional animal ethics committee (IAEC) (Approval no.UIP/IAEC/2014/FEB/12).

### Drugs and chemicals

MTX (Folitrax-15, IPCA Laboratories Limited, Mumbai, India) was acquired from the local market and rutin was procured from Lobachemie Private Limited, Mumbai, India. All other chemicals used were of analytical grade and purchased from Hi-media Laboratories, Mumbai, India.

### Experimental design

Animals were randomly selected and divided into six groups of six animals each and subjected to treatment as depicted in Table 1. The MTX was administered for one week with concomitant administration of rutin (50 and 100 mg/kg, i.p.) [13].

**Table 1:** Effects of rutin on intestinal pH, total acidity, free acidity and CMDI induced by MTX in rats

Groups	Treatment	pH (mEq/l)	Total acidity	Free acidity (mEq/l)	CMDI
Group-I	Sham control (Normal saline, 3.0 ml/kg)	7.15 ± 0.01	11.02 ± 0.61	7.47 ± 0.61	0.33 ± 0.15
Group-II	Toxic control (MTX, 2.5 mg/kg)	6.32 ± 0.04^a^	15.06 ± 0.80^a^	11.46 ± 0.58^a^	4.08 ± 0.05^a^
Group-III	Rutin (50 mg/kg)	7.67 ± 0.03^***a^	11.74 ± 0.69^**^ (22.05 %)	8.45 ± 0.57^***^ (26.20 %)	1.17 ± 0.41^***c^ (58.25 %)
Group-IV	Rutin + MTX(50 + 2.5 mg/kg)	7.44 ± 0.02^***a^	12.68 ± 0.87^***^ (15.74 %)	11.14 ± 0.60^a^ (2.70 %)	1.67 ± 0.52^***a^ (58.32 %)
Group-V	Rutin (100 mg/kg)	7.81 ± 0.01^***a^	11.06 ± 0.38^***^ (26.57 %)	8.70 ± 0.31^***^ (24.01 %)	1.17 ± 0.52^***c^ (58.25 %)
Group-VI	Rutin + MTX (100 + 2.5 mg/kg)	7.21 ± 0.01^***^	12.75 ± 0.85^***^ (15.28 %)	10.13 ± 0.71^b^ (11.52 %)	2.50 ± 0.55^***a^ (37.50 %)

Each group contains six animals. Values are represented as mean ± SEM

Statistical significance compared to toxic control using one-way ANOVA followed by Bonferroni test

(*P < 0.05, **P < 0.01, and ***P < 0.001). Values in parenthesis represent percentage inhibition

Statistical significance compared to sham control using one-way ANOVA followed by Bonferroni test

^c^ *P* < 0.05, ^b^ *P* < 0.01 and ^a^ *P* < 0.001 were considered statistically significant

### Estimation of pH, free acidity and total acidity

After the respective treatments, the animals were euthanized and the intestinal tissues were collected. The intestinal content was collected and evaluated for pH (Hanna Instruments, HI 98107), acidity (total and free), using procedures illustrated previously [14, 15].

### Assessment of colonic mucosal disease index (CMDI)

The colon tissue of approximately 10 cm from the anus was taken, opened longitudinally and washed in normal saline buffer and fixed on the wax block. The scoring was done and appraised as follows. 0 = normal mucosa, 1 = mild hyperemia, no erosion or ulcers on the mucosa surface, 2 = moderate hyperemia, erosion or ulcers appear on the mucosa surface, 3 = severe hyperemia, necrosis and ulcers on the mucosa surface with the ulcerative area less than 40 %, 4 = sever hyperemia, necrosis and ulcers on the mucosa surface with ulcerative area more than 40 % [16].

### Biochemical estimations

 Intestinal tissue (10 % w/v) was homogenized in 0.15 M KCl at 11200 g at 4 °C. The supernatants were figured out for the biochemical changes through TBARS [17], SOD [18], GSH [19], catalase [20] and protein carbonyl [21] using established methods at our laboratory. The tissue supernatant was also manipulated for estimations of interleukin-2 (IL-2) (K0332100P), interleukin-4 (IL-4) (K0332133P) and interleukin-10 (IL-10) (K0332134P) using radioimmunoassay kits (Koma Biotech inc., Seoul, Korea).

### COX and LOX inhibition assay

For determination of the viable anti-inflammatory mechanism, rutin was assayed for COX-1, COX-2 and 15-LOX inhibitory activity using a COX-inhibitor (catalog No.760111) and LOX- inhibitor screening kit (catalogue no-760700); Cayman Chemical Company, USA according to the manufacturer’s instructions. The stock solution of the pantoprazole and rutin were prepared in water for injection and further dilutions were made up to a concentration of 1 μg/ml. Percent inhibition was computed by contemplating the absorbance intensities, deliberated spectrophotometrically with a 96 well plate reader (ALERE Microplate Reader, AM-2100) at 590 nm and 490 nm for COX and LOX respectively.

### Statistical analysis

All data were presented as mean ± SEM and analyzed by one way ANOVA followed by Bonferroni test for the possible significance identification between the various groups. *P < 0.05, **P < 0.01, ***P < 0.001 were considered statistically significant. Statistical analysis was carried out using Graph pad prism software (3.2), San Diego, CA.

## Results

### Effects of rutin on intestinal pH, free acidity, total acidity and CMDI induced by methotrexate in rats

The administration of rutin manifested significant protection against intestinal lesions damaged by MTX. The treatment with rutin elicited noticeable inhibition of free acidity (26.20 %), total acidity (22.05 %) and CMDI (1.16 %) in the experimental animals similar to control (Table 1).

### Effects of rutin on intestinal oxidative damage induced by methotrexate in rats

In MTX treated toxic group, the level of MDA increased, which was subsequently restored after rutin treatment (Table 2). As for protein oxidation, the MTX treatment validated in a comparable increase in protein carbonyl level in the toxic group (42.38 ± 0.35 nanomoles/ml) in comparison to normal (38.41 ± 1.61 nanomoles/ml). The groups treated with rutin restored the level of protein carbonyl to a significant level. Similarly, a decrease in SOD level was observed in MTX treated toxic group (0.29 ± 0.035 SOD/mg of protein) in resemblance to normal control (1.82 ± 0.035 SOD/mg of protein) (Table 2). Rutin restored the levels of SOD in treatment groups. A significant decrease in the enzymatic activity of catalase was observed in toxic control (0.45 ± 0.03 nM of H_2_O_2_/min/mg of protein). It is noteworthy that treatment with rutin restored the catalase activity in treatment groups as well.

**Table 2:** Effects of rutin on intestinal oxidative damage induced by methotrexate in rats

Groups	Treatment	TBARS (nM of MDA/mg of protein)	GSH (mg%, 1 × 10^−4^)	SOD (unit of SOD/mg of protein)	Catalase (nM at H_2_O_2_/min/mg of protein)	Protein carbonyl (nM/ml)
Group-I	Sham control (Normal saline, 3.0 ml/kg)	0.82 ± 0.18	30.63 ± 2.38	1.82 ± 0.035	1.19 ± 0.35	38.41 ± 1.61
Group-II	Toxic control (MTX, 2.5 mg/kg)	5.57 ± 0.12^a^	117.17 ± 1.12^a^	0.29 ± 0.035^a^	0.45 ± 0.03^***^	42.38 ± 0.35
Group-III	Rutin (50 mg/kg)	5.05 ± 0.47^***a^	63.65 ± 1.20^***a^	1.31 ± 0.023^***a^	1.50 ± 0.54	16.36 ± 0.23^***a^
Group-IV	Rutin + MTX (50 + 2.5 mg/kg)	4.49 ± 0.25^***a^	89.48 ± 1.13^***a^	1.58 ± 0.005^***a^	0.40 ± 0.07^**^	27.27 ± 0.91^*b^
Group-V	Rutin (100 mg/kg)	2.87 ± 0.13^***a^	48.76 ± 1.06^***a^	1.55 ± 0.011^***a^	0.78 ± 0.33	24.09 ± 0.46^**a^
Group-VI	Rutin + MTX (100 + 2.5 mg/kg)	2.97 ± 0.21^***a^	51.92 ± 1.70^***a^	2.63 ± 0.046^***a^	1.66 ± 0.48^*^	31.52 ± 5.76

Each group contains six animals. Values are represented as mean ± SEM

Statistical significance compared to toxic control using one-way ANOVA followed by Bonferroni test

*P < 0.05, **P < 0.01, and ***P < 0.001 were considered statistically significant

Statistical significance compared to sham control (Group-I) using one-way ANOVA followed by Bonferroni test

^c^ *P* < 0.05, ^b^ *P* < 0.01 and ^a^ *P* < 0.001 were considered statistically significant

The tissue GSH level was up surged in MTX treated toxic group (117.17 ± 1.12 mg %) in context to normal control (30.63 ± 2.38 mg %). When rutin was given, the GSH level was restored.

### Effects of rutin on intestinal cytokines induced by methotrexate in rats

The tissues were also contemplated for the presence of inflammatory markers like IL-2, IL-4 and IL-10. IL-2 is a proinflammatory marker whereas IL-4 and IL-10 are anti-inflammatory cytokines. The MTX treated toxic group perceived in a comparable upsurge in the levels of interleukins, and rutin restored the same (Table 3).

**Table 3:** Effects of rutin on intestinal cytokines induced by methotrexate in rats

Groups	Treatment	IL-2 (pg/ml)	IL-4 (pg/ml)	IL-10 (pg/ml)
Group-I	Sham control (Normal saline, 3.0 ml/kg)	398.10 ± 5.32	162.18 ± 8.79	63.09 ± 5.51
Group-II	Toxic control (MTX, 2.5 mg/kg)	671.93 ± 56.65^a^	213.79 ± 7.64^a^	88.57 ± 3.45^a^
Group-III	Rutin (50 mg/kg)	174.15 ± 8.20^***a^	193.50 ± 1.47^***a^	104.05 ± 0.65^**a^
Group-IV	Rutin + MTX(50 + 2.5 mg/kg)	233.52 ± 14.69^***a^	117.29 ± 0.023^***a^	63.21 ± 2.96
Group-V	Rutin (100 mg/kg)	178.72 ± 3.28^***a^	198.26 ± 7.76^***a^	122.11 ± 1.88^***a^
Group-VI	Rutin + MTX (100 + 2.5 mg/kg)	272.02 ± 15.00^***a^	179.24 ± 1.57^***^	96.99 ± 1.50^***a^

Each group contains six animals. Values are represented as mean ± SEM

Statistical significance compared to toxic control using one-way ANOVA followed by Bonferroni test

*P < 0.05, **P < 0.01, and ***P < 0.001 were considered statistically significant

Statistical significance compared to normal control (Group-I) using one-way ANOVA followed by Bonferroni test

^c^ *P* < 0.05, ^b^ *P* < 0.01 and ^a^ *P* < 0.001 were considered statistically significant

### Effects of rutin on activities of COX-1, COX-2 and 5-LOX

The Rutin was found to inhibit the enzymatic activity of COX-1, COX-2 and 15 LOX with 75.63, 81.00 and 80.43 % inhibition respectively (Table 4).

**Table 4:** Effects of rutin on activities of COX-1, COX-2 and 5-LOX

Drug	COX-1 (% Inhibition)	COX-2 (% Inhibition)	15-LOX (% Inhibition)
Rutin (1 μg/μl)	75.63 ± 6.48	81.00 ± 3.96	80.43 ± 5.19

Results are expressed as Mean ± SEM (*n* = 3)

## Discussion

MTX is an antimetabolite and antifolate agent which competitively inhibits the binding of folic acid to its cognate enzyme DHFR [22]. MTX was originally refined and continues to be used for chemotherapy either alone or in combination with other agents [23]. The use of MTX is often times assigned with intestinal toxicities along with cardiotoxicity, nephrotoxicity and hepatotoxicity [24, 25]. To minimize the side effects in patients undergoing chemotherapy, it is important to curtail mucosal damage and prompt up the tissue repair. The antecedent studies have accumulated the concurrence of ROS in the pathogenesis of MTX toxicity. Various research groups had exploited the pathway for ROS to counteract such toxicities, and the same was contemplated in the present experiment through concomitant therapy of rutin [26].

Treatment with rutin evidenced a dose dependent preservation against the intestinal toxicity when ascertained through physiological parameters. The rutin treatment manifested compelling rise in intestinal pH with decrease in the total and free acidity. The CMDI score in the toxic control was also scaled down momentously by rutin in a dose dependent manner suggesting a positive modulation by rutin. Prima facie the rutin was observed to have marked assurance against the MTX induced intestinal toxicity, and the same could be accredited to the antisecretory potential of rutin [17].

To further investigate, we figured out the effect of rutin on enzymatic antioxidant defense along with markers of lipids and proteins peroxidation. Increased production of MDA and protein carbonyl was illustrated in the MTX treated group, which is in corroboration with the previous studies [27, 28]. Body cells and tissues are in endless threat by the free radicals, which are propagated either through normal oxygen metabolism or induced by exogenous damage (MTX in present case) [29–31]. The mechanism/s or sequence/s of events associated with the free radicals bracketed cellular dysfunction is not fully understood, but lipid and protein peroxidation are the important markers for this [32]. The protein and lipid adjustment elected by the direct oxidative stress on amino acid residues and phospholipids can link to the formation of carbonyl derivatives and MDA, which are extensively used as a marker for oxidative stress [21]. In the present experimental condition, MTX proclaimed significant increase in the levels of protein carbonyl along with MDA, and rutin subsequently helped to restore the same in a dose dependent manner.

GSH plays an important role in protecting tissues from per oxidative attack and is perceived as a first line defense against the ROS attack. The previous report suggests that the tissue GSH levels are subsided as a repercussion of peroxidative attack [33]. Nonetheless, we contemplated marked increase in the tissue GSH levels after MTX toxicity, and the same could be attributed to the marked up biogenesis of GSH as a feedback, in consequence to overwhelming ROS generation. Rutin was contemplated to restore the GSH levels in dose dependent manner and the same could be attributed to decrease in oxidative stress. The above discussion clearly acclaims the participation of ROS in MTX toxicity and its affray by rutin.

The SOD and catalase integrate a major supportive team of defense against ROS. SOD catalyses the scavenging of the superoxide radical leading to the formation of hydrogen peroxide, which is afterwards catabolised by catalase to water and molecular oxygen. Both the enzymes work in tandem to affray the ROS [34, 35]. In the present experiment, a significant decrease in the enzymatic activity of SOD and catalase was prevalent in the MTX treated group, which could be accredited to the up turned oxidative stress. Rutin implemented a conspicuous protection against the MTX induced intestinal toxicity in a dose dependent manner. After going through the biological makers of the oxidative stress, the authors are in a strong opinion that rutin can flapdown the deleterious effects associated with the MTX chemotherapy.

The precedent studies have also suggested the immune dysfunction coupled with the MTX toxicity [36]. Henceforth, we in quested the levels of anti-inflammatory (IL-10) and immunoregulatory (IL-2 and IL-4) cytokines after the concomitant administration of rutin. The IL-2 and IL-4 are the signaling molecules in the immune system. The IL-2 regulates the activity of white blood cells and IL-4 participates in the differentiation of naive helper T cells (Tho cells) to Th2 cells [37]. Both IL-2 and IL-4 are produced either normally or as a consequence of activation by T cells during an immune response [38, 39]. We ascertained a momentous increase in the activity of IL-2 and IL-4 after the MTX therapy and are of the opinion, that the utmost immunocompromised state after MTX could have actuated a feedback mechanism. Nonetheless, the levels of IL-2 and IL-4 were somewhat restored after the rutin administration, implying the redeemed immunological status. When observed for IL-10 (anti-inflammatory cytokine) the MTX administration perceived momentous decrease and the same was restored to normal after low dose rutin. The MTX administration was clearly suggestive of immunocompromised status with pronounced inflammation and the same was restored to normal with subsequent administration of rutin.

The free radical scavenging activity of rutin and its metabolite quercetin is a well studied phenomenon. Both rutin and quercetin can suppress the free radical generation at three stages: formation of superoxide ion, generation of hydroxyl (cyprohydroxy) radical in fenton reaction and formation of lipid peroxy radicals [40]. Nonetheless, the mechanism beneath the anti-inflammatory potential of rutin is unexplored/not reported until now and to inquest the same we enumerated the effect of rutin on COX and LOX enzymes in vitro. The results helped us to derive that; rutin by inhibiting the COX/LOX pathway of AA metabolism can curb the inflammatory reaction as conceded with MTX. It would be pertinent to mention that dual inhibitors of AA metabolism have manifested an excellent preclinical gastrointestinal pharmacological safety (Argentieri et al., 1994). Moreover, recent findings from phase II and III studies of the clinical development of licofelone (dual inhibitor of AA metabolism) indicate that licofelone has an excellent gastrointestinal safety, which is better than conventional NSAIDs [41]. It is to be noted that rutin when given independently could afford significant rise in the intestinal pH, total acidity and free acidity, which could be accredited to the formation of polar metabolites of rutin in the intestine [42]. It is to be noted further that MTX is excreted (90 %) unchanged in the urine through combination of glomerular filtration and active tubular secretion and changes/decrease in renal excretion can delay elimination and lead to severe myelosuppression [43]. MTX is often prescribed with biocarbonate to maintain the pH of above 6.5 and subsequent excretion. The increase in intestinal pH by rutin therefore may afford a facilitated excretion of MTX and thus could eliminate the use of biocarbonates in therapeutic regimes along with chances of myelosuppression.

## Conclusion

Henceforth, one can derive that the rutin exhibits significant physiological, biochemical and immunological protection against MTX induced intestinal toxicity in a dose dependent manner and can be advocated as an adjuvant therapy in MTX chemotherapy to countervail the deleterious effects. The free radical scavenging, immunoregulatory and dual inhibitory potential of AA metabolism could be considered as the possible mechanisms for the actions of rutin as observed in the present experiment.

## Abbreviations

AA: arachidonic acid
CMDI: colonal mucosal disease index
COX: cyclooxygenase
DTNB: 5,5’-Dithiobis-(2-Nitrobenzoic Acid)
GSH: glutathione
IL: interleukin
LOX: lipoxygenase
MDA: malondialdehyde
MTX: methotrexate
ROS: reactive oxygen species
SEM: scanning electron microscope
SOD: superoxide dismutase
TBARS: thiobarbituric acid reactive substance
TCA: trichloro acetic acid
